# EMG feedback outperforms force feedback in the presence of prosthesis control disturbance

**DOI:** 10.3389/fnins.2022.952288

**Published:** 2022-09-20

**Authors:** Jack Tchimino, Jakob Lund Dideriksen, Strahinja Dosen

**Affiliations:** Neurorehabilitation Systems, Department of Health Science and Technology, Aalborg University, Aalborg, Denmark

**Keywords:** closed-loop control, somatosensory feedback, EMG feedback, vibrotactile stimulation, prosthetic hand, grasping force control, disturbance

## Abstract

Closing the prosthesis control loop by providing artificial somatosensory feedback can improve utility and user experience. Additionally, closed-loop control should be more robust with respect to disturbance, but this might depend on the type of feedback provided. Thus, the present study investigates and compares the performance of EMG and force feedback in the presence of control disturbances. Twenty able-bodied subjects and one transradial amputee performed delicate and power grasps with a prosthesis in a functional task, while the control signal gain was temporarily increased (high-gain disturbance) or decreased (low-gain disturbance) without their knowledge. Three outcome measures were considered: the percentage of trials successful in the first attempt (reaction to disturbance), the average number of attempts in trials where the wrong force was initially applied (adaptation to disturbance), and the average completion time of the last attempt in every trial. EMG feedback was shown to offer significantly better performance compared to force feedback during power grasping in terms of reaction to disturbance and completion time. During power grasping with high-gain disturbance, the median first-attempt success rate was significantly higher with EMG feedback (73.3%) compared to that achieved with force feedback (60%). Moreover, the median completion time for power grasps with low-gain disturbance was significantly longer with force feedback than with EMG feedback (3.64 against 2.48 s, an increase of 32%). Contrary to our expectations, there was no significant difference between feedback types with regards to adaptation to disturbances and the two feedback types performed similarly in delicate grasps. The results indicated that EMG feedback displayed better performance than force feedback in the presence of control disturbances, further demonstrating the potential of this approach to provide a reliable prosthesis-user interaction.

## Introduction

Upper limb loss has profound and lasting effects on the quality of life of those affected (Shahsavari et al., 2020). Amputees face significant challenges regarding the execution of daily tasks (Antfolk et al., 2013), returning to the workplace after their injury (Pomares et al., 2020; Shahsavari et al., 2020) and participating in social activities (Kristjansdottir et al., 2020; Shahsavari et al., 2020), while also potentially suffering from phantom limb pain (Esquenazi, 2002). Nowadays, myoelectric prostheses offer substantial restoration of lost hand functions; however, despite the ever-growing sophistication of these devices, users often find their control cumbersome and have difficulty accepting them as an integral part of their anatomy, resulting in high abandonment rates (Salminger et al., 2020).

Most commercial myoelectric prostheses are controlled using a direct proportional approach (Peerdeman et al., 2011; Roche et al., 2014), wherein the myoelectric signal is directly mapped to the velocity of prosthesis movement. It has been shown in the literature that closing the control loop by means of artificial somatosensory feedback enhances the performance and user experience of such control schemes, offering more precise control and sense of embodiment (Antfolk et al., 2013; Markovic et al., 2018a; Wilke et al., 2019; Bensmaia et al., 2020). Normally, somatosensory feedback is implemented by reading data from sensors embedded in prosthesis and conveying this information to the user by stimulating their residual limb mechanically or electrically (Jabban et al., 2022). Mechanical stimulation is delivered to the skin using vibration motors, rotational and linear actuators (Fu et al., 2019; Thomas et al., 2019; Abd et al., 2022). Alternatively, electrical stimulation can be delivered to the skin via non-invasive (Isakovic et al., 2019; Vargas et al., 2022) or invasive means, through peripheral nerve or even brain interfaces (Pasluosta et al., 2018). Different feedback variables have been used in the past to provide artificial exteroceptive and proprioceptive feedback, the most common of which was the grasping force (Brown et al., 2015; Fu and Santello, 2018; Fu et al., 2019; Vargas et al., 2022). More recently, electromyography (EMG) feedback, where the user is informed of the level of their muscle contraction (Dosen et al., 2015b; Schweisfurth et al., 2016; Engels et al., 2019; Tchimino et al., 2021) has been tested, demonstrating that this approach can facilitate predictive control of prosthesis grasping.

However, it is well known that the efficiency of such control strategies in clinical settings can be substantially impaired by the various sources of disturbance that perturb the control signals, which are largely absent from the rigorously controlled laboratory environment (Yang et al., 2019). A disturbance in the control signal will manifest itself as a different motor behavior in the prosthesis, posing a risk of damage to grasped objects (e.g., unintentional slipping or breaking). While the impact of such disturbances and the users’ capability to adapt and compensate for them has been tested (Hahne et al., 2017), such an assessment has been seldomly performed regarding differing feedback approaches. In general, despite many feedback methods having been presented so far, using different feedback variables, stimulation techniques, and encoding methods, such approaches are rarely compared (Engels et al., 2019; Marasco et al., 2021). Such a comparison, however, is important, as it could be used to critically inform the selection and implementation of feedback interfaces.

An intrinsic characteristic of closed-loop control is that it should be more robust regarding disturbances and noise injected into the system. However, in the context of prosthesis control, the users’ ability to react and compensate for the disturbance is likely affected by the specific type of feedback they receive. For example, EMG and force feedback are fundamentally different, therefore, it is reasonable to assume that the effectiveness of disturbance mitigation will also differ between them. In the case of EMG feedback, the user receives online information about their muscle contraction (prosthesis control input), even while the hand is in motion and not in contact with an object. This allows predictive force control, since the user can modulate or correct the generated EMG level even before the prosthesis grasps an object. Conversely, in the case of force feedback, the user must wait until the prosthesis makes contact with an object to assess the generated force and correct if necessary. Hence, it can be expected that EMG feedback would facilitate the compensation of control disturbances. In this case, the feedback would provide information on the control signal, thereby allowing the user to notice if the signal is disturbed and then modulate their muscle contraction in time, before the hand closes around the object and an erroneous force is applied.

The aim of this study was, therefore, to assess and compare the performance of prosthesis control when using EMG versus force feedback in the presence of control disturbances. The subjects were asked to perform a force matching task using a sensorized myoelectric prosthesis over a number of trials. In some of these trials, the myoelectric signal was amplified or attenuated, unbeknownst to the subjects.

We hypothesized that the subjects would perform better when using EMG feedback. More specifically, we assumed that they would be able better compensate for the disturbances as soon as they appeared; and that they would need fewer attempts to adapt to the disturbance in case they failed to compensate immediately. Lastly, a shorter completion time was expected when EMG feedback was used thanks to the real-time feedback flow.

## Materials and methods

### Subjects

Twenty healthy able-bodied subjects (28.5 ± 4.4 years), with no prior experience in myoelectric control, participated in this study. The subjects were divided into two groups one of which performed the experiment using EMG feedback and the other using force feedback. In addition, one subject with left transradial amputation performed the experiment in both feedback conditions in two separate sessions. The amputee was 49 years old, had lost their arm 10 years prior and had been using a simple myoelectric prosthesis for 8 years. Prior to the present experiment, the amputee did not use a prosthesis equipped with artificial somatosensory feedback. The subjects signed an informed consent form before commencing the experiment, which was approved by the Research Ethics Committee of the Nordjylland Region (approval number N-20190036).

### Experimental setup

**FIGURE 1:**
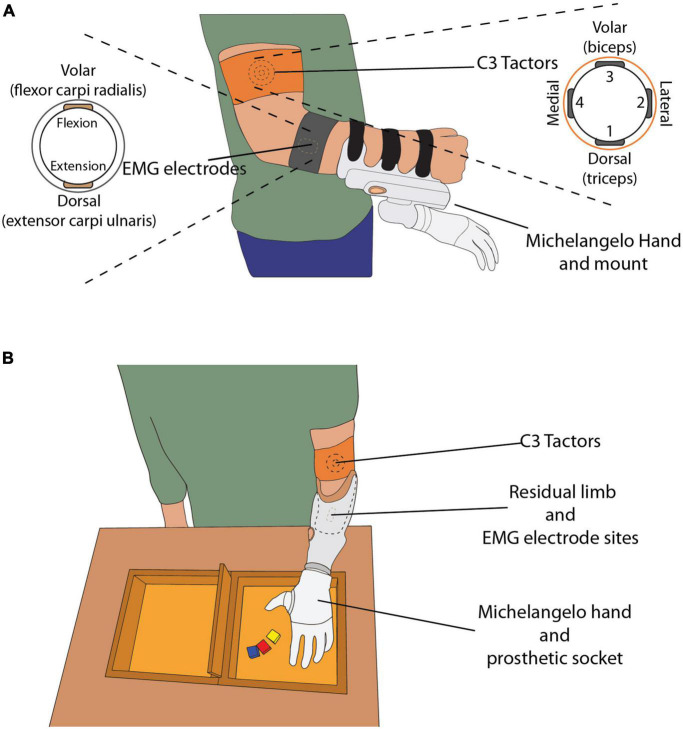
**(A)** The able-bodied subject wearing C3 tactors, EMG electrodes and the mount with the Michelangelo hand attached. The drawings on the left and right display the placement of the electrodes and tactors around the subject’s arm, respectively. **(B)** The amputee subject wearing the custom-made socket, with the EMG electrodes embedded in the socket, and C3 tactors placed on their left upper arm. The box and blocks setup is placed on the table in front of the subject.

The experimental setup for able-bodied subjects comprised the following components: (1) a multifunctional myoelectric prosthetic hand (Michelangelo hand, Otto Bock, Duderstadt, Germany), with proprietary controller, two dry EMG electrodes with embedded amplifiers (13E200, Otto Bock, Duderstadt, Germany) and a USB Bluetooth dongle, (2) four C3 vibrotactors and a control unit (Engineering Acoustics Inc., Casselberry, FL, United States), (3) a standardized box-and-blocks test, (4) a standard laptop (Lenovo ThinkPad P52, Intel Core i7 @2.60 GHz, 32 GB RAM), running Windows 10 Professional and an 18″ computer monitor. The able-bodied subjects carried the prosthesis on their right forearm using a specially made 3D printed mount (Figure 1A) and their wrist was immobilized with a thermoplastic orthopedic splint (ORLIMAN), to enforce isometric muscle contractions. The prosthesis was connected to the laptop via Bluetooth, using the USB dongle, while the tactor control unit was connected to the laptop via USB. The program that controlled the prosthesis and generated vibrotactile feedback was implemented in MATLAB Simulink 9.3, using the toolbox for closed-loop human-manual control (Dosen et al., 2015a).

The prosthesis controller sampled the EMG, computed the root mean square (RMS) in 100-ms windows, and sent the data to the laptop at a sampling frequency of 100 Hz. The electrodes were placed on the skin above the flexor carpi radialis and extensor carpi ulnaris muscles (Figure 1A), identified by palpation. The electrodes were attached to the skin with medical adhesive tape and an elastic band was placed around the forearm to ensure good electrode-skin contact and avoid electrode shift. If the EMG signal quality was poor, the electrodes were removed, a small amount of conductive gel was applied onto the skin to improve the electrode-skin interface and the electrodes were replaced.

The amputee subject was fitted with a custom-made socket, to which the Michelangelo hand was connected (Figure 1B). The socket was designed so that the electrodes were placed in the same position as in the socket normally used by the amputee.

The C3 tactors produce vibrations perpendicular to the skin, with adjustable gain and frequency. In this experiment, the frequency was set at 230 Hz, which corresponds to the maximum sensitivity of the Pacinian corpuscles (Gilman, 2002). The four tactors were placed equidistantly around the upper arm (Figure 1), approximately 5 cm proximal to the elbow, and were held in place with an elastic band. The biceps brachii was used as an anatomical landmark for the placement of the tactors. The tactors’ gains were adjusted based on the sensation threshold (ST), determined for each subject (see section “Experimental protocol”). The tactors were placed around the upper arm of the amputee in the same configuration as in the able-bodied subjects.

The subjects stood in front of a desk, wearing the setup on their arm (Figure 1). The height of the desk was adjusted so that the subjects could execute the task comfortably, without fatiguing by lifting their arm too high. The box and blocks setup was placed on the desk in front of them and the computer monitor was positioned approximately 50 cm away from them.

The Michelangelo prosthesis supports velocity control over two degrees of freedom, namely hand opening/closing using two grasp types (palmar and lateral) and wrist rotation. In this experiment, the hand was configured to only open and close in palmar grasp. The command input to the prosthesis was a normalized myoelectric signal (ranging from 0 to 1). While the hand was in motion, this signal was proportional to the closing velocity and the grasping force generated upon contact (i.e., stronger contraction, faster closing, and higher force). After contact, a further increase in the prosthesis command input proportionally increased the force. The force was measured using sensors embedded in the hand, transmitted to the laptop at 100 Hz and normalized to the maximum force produced by the hand when it closed at the highest velocity. Importantly, the prosthesis was non-backdrivable and after contact responded only to an increase in the command input, while the grasping force remained unchanged if the myoelectric signal decreased. For instance, the subjects could relax their muscles and the prosthesis would still maintain the generated grasping force.

### Closed-loop prosthesis control

**FIGURE 2:**
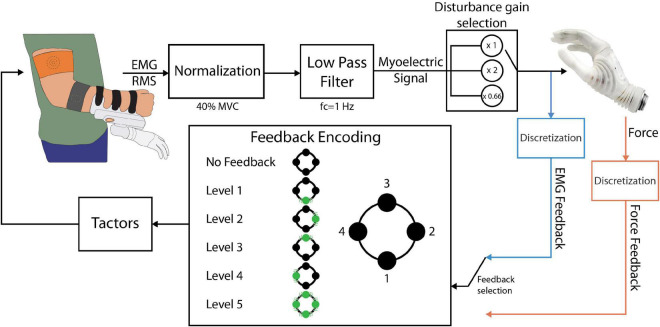
The closed-loop prosthesis control scheme. The electrodes outputted the RMS of the EMG, which was low-pass filtered and normalized to 40% of the MVC, resulting in the myoelectric (ME) control signal. The ME signal was sent to the prosthesis as a closing velocity or opening command. If EMG feedback was used, the ME signal was discretized into levels and conveyed back to the subject through the tactors. If force feedback was used, the force was discretized and fed back to the subject using the same encoding. The position and numbering of the tactors in this diagram is consistent with the one shown in Figure 1.

The closed-loop control scheme implemented in this study is shown in Figure 2. The EMG RMS was normalized to 40% of the maximum voluntary contraction (MVC) and then low pass filtered using a second-order Butterworth filter with a cutoff frequency of 1 Hz. These parameter values were selected based on the results of our previous study (Tchimino et al., 2021), investigating the optimal calibration of EMG feedback. The normalized flexor signal was then mapped to the normalized closing velocity, where 0 indicated no movement and 1 corresponded to the maximum closing speed of the prosthesis. Hand opening was controlled using a simpler scheme, as it was not relevant for the task (see section “Experimental protocol”). Specifically, when the normalized extensor signal surpassed a threshold of 0.4, the hand opened to its full aperture. The grasping force measured by the embedded force sensors was also normalized (0 – no force, 1 – maximum force).

Two feedback types were implemented: EMG and force feedback. Each subject received one of the two feedback types, depending on the experimental group to which they were assigned. For the feedback generation, the myoelectric and force signals were divided into five intervals and the feedback conveyed to the subject the discrete level of the signal, i.e., the interval in which the EMG/force lay at any moment (Figure 2). The threshold values for the intervals were {0.1, 0.2, 0.4, 0.65, and 0.95}. The thresholds were selected so that levels 1 to 4 were of increasing size to mitigate the larger variability of EMG arising from stronger muscle contractions (Ninu et al., 2014; Dosen et al., 2015b; Schweisfurth et al., 2016; Tchimino et al., 2021).

The feedback encoding scheme is shown in Figure 2. When the feedback signal (EMG or force) was in the dead zone (<0.1), no tactors were active. Each subsequent level was indicated by activating the corresponding tactor, while level 5 activated all four tactors simultaneously. Hence, the subject received vibrations that moved from the dorsal aspect of their upper arm to the lateral, volar, and medial aspects and finally all around the upper arm, as the level of the feedback signal increased. Discrete feedback in combination with spatial encoding has been shown to be an easy to understand, effective approach for closed-loop myoelectric control (Witteveen et al., 2012; Schweisfurth et al., 2016; De Nunzio et al., 2017; Markovic et al., 2018a,b; Tchimino et al., 2021).

**FIGURE 3:**
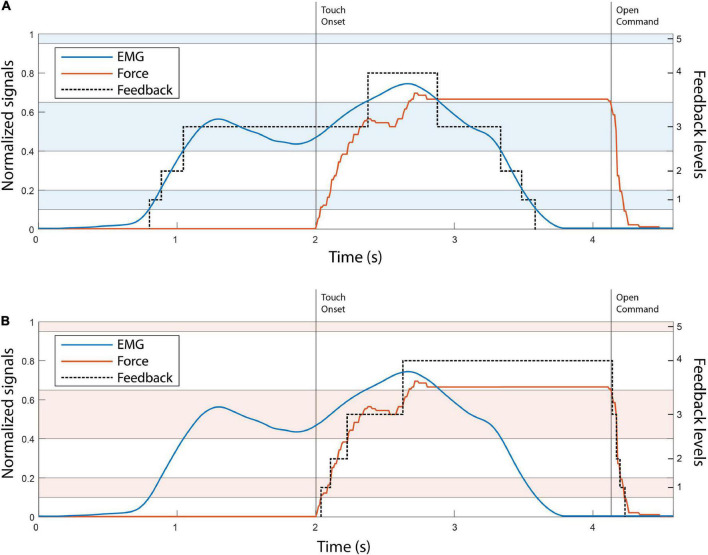
The illustration of EMG **(A)** and force **(B)** feedback. The EMG and force traces in both panels A, B are denoted with blue and red, respectively. The black dotted line in both plots indicates the feedback level. The alternating color bands are the EMG and force ranges. The touch onset and the moment that the open command was sent to the hand are denoted with vertical black lines. Note that levels 1–4 are of progressively larger size, to account for the higher EMG variability in stronger contractions.

The control of the Michelangelo hand using EMG and force feedback is illustrated in Figures 3A,B, respectively. In the case of EMG feedback, the subject received vibration as soon as they activated their muscles, and the hand started moving (Figure 3A). This allowed them to adjust their contraction level while the prosthesis was still in motion. Due to the proportional relation between the myoelectric signal, prosthesis closing velocity and grasping force, the subjects knew that the grasping force level produced by the prosthesis would correspond to the EMG feedback level that they received. Conversely, force feedback was only activated after the prosthesis had closed and applied force onto the object.

### Disturbance scheme

The effect of control perturbations in myoelectric systems has been investigated in the past. Examples include the introduction of delays in the feedback delivery (Cipriani et al., 2014), injecting additive noise in the myoelectric signal (Hahne et al., 2017), artificial errors in visual feedback (Johnson et al., 2017), and electrode shift (Prahm et al., 2019). In the present study, the control disturbance was implemented by multiplying the prosthesis control signal by a gain, unbeknownst to the subjects (Figure 2, “disturbance gain selection” block) (Cipriani et al., 2014). Similarly to an approach implemented in Earley et al. (2017) and Earley et al. (2021), the gain of the myoelectric signal was doubled or reduced by 33% at the beginning of a disturbed trial. When the gain was doubled (high-gain disturbance), the system became more sensitive with respect to the nominal condition and the same muscle contraction now generated a stronger myoelectric signal. This, in turn, produced a faster closing of the prosthesis and a higher grasping force. Reducing the gain (low-gain disturbance) had the inverse effect, i.e., slower closing and lower force, with a larger contraction required to reach a specific EMG and force level. The subjects were not informed beforehand that any of the trials would be disturbed, as explained in the next section.

### Experimental protocol

The subjects were first asked to rest their arm for 10 s while the baseline recording was performed. The mean value of the recorded signal (EMG RMS) was then subtracted from subsequent measurements. Afterward, the MVC was recorded for both flexors and extensors. The subject contracted each muscle maximally in three intervals of 5 s. The mean value of the recorded signal was computed in each interval and the MVC for each muscle was defined as the average of these three values.

Next, the sensation thresholds (ST) for the four C3 tactors were determined. Each tactor was activated in sequence and its gain, which corresponded to the vibration intensity, was slowly increased in steps of 2%. The gain at which the subject reported that they started feeling a vibration was defined as the ST. The gain of each tactor was subsequently set to ST + 0.4 × (MAX-ST), where MAX is the maximum gain (Tchimino et al., 2021). This value was selected as it elicited clear and localized vibrations, which were not intrusive.

The feedback training was then conducted, wherein the five levels of vibrotactile feedback were presented sequentially to the subjects, while the experimenter verbally indicated which tactors were active. A short session of reinforced learning followed, where the stimulation patterns for levels 1–4 were delivered in a pseudorandom order and the subject was asked to identify each pattern. If the subject reported the wrong pattern, the experimenter provided the correct answer. Each pattern was presented 10 times in total. In the validation phase, the test was repeated, without the experimenter providing verbal feedback. If the identification rate was above 90%, the feedback training was deemed successful and if not, the procedure was repeated. Most subjects achieved a success rate of above 90% immediately after the first run, indicating that the feedback encoding was indeed simple to learn and interpret (Tchimino et al., 2021).

The subjects then performed the main experimental task. They used the prosthesis equipped with the vibrotactile feedback to perform a functional task inspired by the commonly used box and blocks test (Hebert and Lewicke, 2012; Raveh et al., 2018). The task included grasping wooden blocks with the prosthesis and transporting them from one compartment to the other (e.g., from the left to the right compartment). Once ten blocks were moved, the direction was changed (e.g., from the right to the left compartment). However, in this “sensorized” version of the test, the subjects were asked to grasp each block by exerting a specific grasping force level, indicated on the computer screen. The desired force levels were 2 and 4, which denoted delicate and power grasping, respectively. If the generated force level in a specific trial did not correspond to the target level, the subject repeated the trial. The experimenter also clarified that the forces could only be corrected upwards but not downwards (as the prosthesis was non-backdrivable).

When a trial was successful, a high-frequency beeping sound was generated by the computer. A lower-frequency sound indicated that the subject had failed, and the trial had to be repeated. The subjects were allowed a maximum of 10 attempts to successfully complete each trial. If they were successful in a smaller number of attempts or if they were still unsuccessful in their 10th attempt, they proceeded to the next trial. All subjects managed to complete all the trials within the 10-attempt limit.

The subjects first performed a 100-trial training session (50 trials for each of force levels 2 and 4), to familiarize themselves with the setup and the task. At this stage, they could see their EMG signals on the monitor, as well as their current target force level, generated force, EMG level and remaining number of attempts. Before commencing, the experimenter verbally explained the setup, the prosthesis control and feedback scheme, and the task to be performed. Initially, the subjects were asked to perform the task while seated and with the prosthesis placed on the desk, so that they could focus on familiarizing themselves with the feedback. After approximately 25 successful trials, the prosthesis was mounted onto the subjects’ arm and the subjects were asked to stand in front of the desk, facing the box and blocks kit and the monitor. They then performed the experimental task until they reached a total of 100 training trials.

After that, the subjects performed the main experiment, comprising three sets of 100 trials. Each set was performed in batches of 2 min, until a total of 100 trials was completed. There was a pause of approx. 30 s between the batches and a longer break between the sets. During the trials, the subjects could only see their target force, remaining attempts and a timer counting to zero from 120 s on the monitor. They were also instructed to complete as many successful trials as possible within a 2-minute batch. The time constraint was introduced to promote natural and intuitive control, while generating consistent movement profiles (Earley et al., 2017).

The first 100-trial run always included only non-disturbed trials to ensure that the subject was well trained in using the feedback and accomplishing the task in the nominal condition. The second and third runs included 30 disturbed trials (15 in each of the target levels) at a single disturbance gain (random order across the two sets). The order of the trials was pseudorandomized, with a constraint that two consecutive disturbed trials were separated by at least one non-disturbed trial. The sequence of the two disturbance gains was randomized among subjects, to minimize any training effects.

The able-bodied subjects performed the task with one of the feedback types (EMG or force), depending on the group to which they were allocated, while the amputee performed the experiment using both types. The amputee performed the two experimental sessions two days apart, first with force, then with EMG feedback. The experimental sessions lasted between 2 and 2.5 h.

### Data analysis

The outcome measures were: (1) the first attempt success rate (SR) defined as the percentage of trials successful in the first attempt, (2) the average number of attempts in the trials that were unsuccessful in the first attempt, and (3) the average completion time of the successful (last) attempt in each trial. The latter was defined as the time from the moment the myoelectric control signal crossed the dead zone until the moment the maximum force was applied. The outcome measures were computed separately for the two types of disturbed trials (high- and low-gain disturbance), as well as for the non-disturbed trials.

The SR was deemed the most important measure, since an erroneous force potentially results in slippage or breakage of an object. In disturbed trials, this metric can be regarded as a measure of the subjects’ *reaction* to the disturbance, i.e., their ability to compensate the disturbance as soon as it appears (in the first attempt).

The average number of attempts in trials that were unsuccessful in the first attempt is an indication of how effectively the feedback assisted the subjects in adjusting their control input (muscle contraction) across attempts. In disturbed trials, therefore, this can be regarded as a measure of *adaptation* to the control disturbance.

The metrics used in the analysis were computed for each subject and each gain using 30 disturbed trials at that gain. The baseline metrics were also computed, using 100 initial, non-disturbed trials. The results of the amputee subject were interpreted independently and were not considered in the statistical analysis.

Outliers were defined as values above or below 1.5 times the upper and lower quartiles, respectively. The data was tested for normality using the Lilliefors test. The outcome measures were compared between the two feedback types, using an unpaired *t*-test or a Mann-Whitney test, depending on the outcome of the normality test. The statistical toolbox of MathWorks MATLAB 2021a and IBM SPSS 27 were used for the statistical analysis. The results are reported in the text as “median {interquartile range}” and the threshold for significance was set at *p* < 0.05.

## Results

**FIGURE 4:**
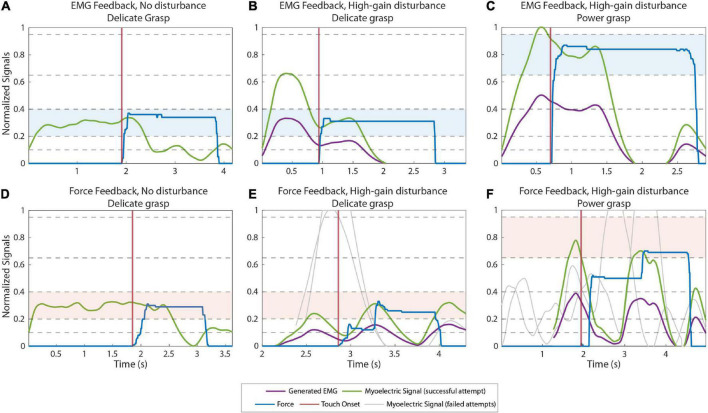
Example myoelectric (ME) and force traces recorded during normal **(A,D)** and high-gain disturbed **(B,C,E,F)** trials when using EMG feedback **(A–C)** and force feedback **(D–F)**. The green and blue lines correspond to the ME signal and the force applied in the last (successful) attempt in a trial, respectively. The purple lines are the ME signals produced by the subjects before the signal is multiplied by the disturbance gain. The gray lines are the ME signals generated in previous attempts of the same trial. The ME signals of all attempts are aligned on their respective contact points (when the prosthesis contacts the object), denoted by the vertical red lines. The target levels in each trial are highlighted with blue **(A–C)** and red **(D–F)** colored bands.

Figure 4 displays the EMG and force traces in normal and disturbed trials with EMG and force feedback. In normal trials (plots A and D), the subjects produced an appropriate muscle contraction (level 2), which generated the desired force (also level 2) once the prosthesis closed around the object, accomplishing the task in a single attempt.

In the high-gain disturbed trials with force feedback (plot E, delicate grasping and plot F, power grasping), the subjects needed multiple attempts to adapt to the disturbance and complete the trial. With respect to the nominal case, the high gain increased the amplitude of the myoelectric signal generated in response to the same muscle contraction. Therefore, the myoelectric signals (gray lines) generated by the subjects were initially too high, in fact saturated, for both delicate (plot E) and power grasping (plot F). Consequently, the generated force overshot the target (object “broken”). In subsequent attempts, the subjects gradually reduced their contraction levels, finally generating the appropriate contraction (green line), to reach the target force in the third attempt.

Conversely, with EMG feedback (Figures 4B,C) the subjects received online information about the generated control signal. Therefore, they immediately compensated for the overshoots caused by the high-gain disturbance by decreasing the muscle contraction with respect to that used in the nominal trials. Hence, they performed the task successfully already in the first attempt.

**FIGURE 5:**
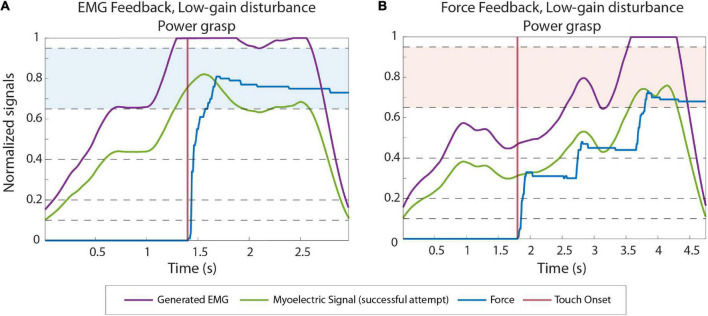
Example EMG and force traces recorded during low gain disturbed trials from subjects using EMG **(A)** and force feedback **(B)**. The green and blue lines correspond to the myoelectric (ME) signal and the force applied in the last (successful) attempt in a trial, respectively. The purple lines are the ME signals produced by the subjects before the signal is multiplied by the disturbance gain. The contact points (when the prosthesis contacts the object) are denoted by the vertical red lines. The target levels in each trial are highlighted with blue and red colored bands.

Figures 5A,B illustrate the power grasping trials with a low gain disturbance using EMG and force feedback respectively. In both cases, the subjects were successful in the first attempt. With reduced gain, the prosthesis closes more slowly for a given muscle contraction, creating a smaller grasp force. When using force feedback, therefore, the subjects initially undershot the force. After the hand made contact with the object, the subject received the feedback, realized that the force was too low and increased the muscle contraction until the desired force was achieved, resulting in a prolonged task duration (completion time of 3.85 s). With EMG feedback, on the contrary, the subject reacted similarly as in the high-gain case. They could immediately “feel” (via feedback) that the nominal contraction was not enough and then increased their contraction to level 4, effectively compensating for the decrease in gain. The task was, therefore, accomplished without force corrections and, hence, in a shorter time (completion time of 1.68 s).

Regarding the results in non-disturbed trials, EMG feedback outperformed force feedback slightly but significantly in terms of SR in power grasping (median {IQR} of 76% {10} against 71% {2.6}, *p* = 0.035) and number of attempts in delicate grasping (2.16 attempts {0.25} against 2.5 attempts {0.13}, *p* = 1.9e-6). However, force feedback resulted in significantly shorter completion time in delicate grasping (1.95 s {0.44} for force, against 2.37 s {0.46} for EMG feedback, *p* = 0.04). In all other cases, the difference in performance between the two feedback types was not significant.

**FIGURE 6:**
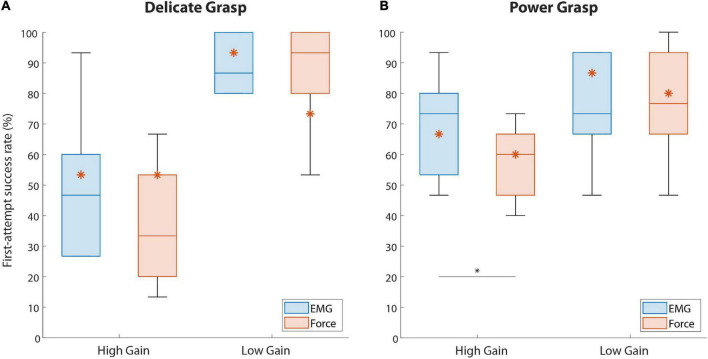
The percentage of trials successful in the first attempt (first-attempt SR), for both force targets. Boxplots **(A,B)** correspond to delicate and power grasps, respectively. The blue and red boxplots represent the summary performance with EMG and force feedback, respectively. The orange asterisks denote the performance of the amputee subject. The boxplots indicate the median (horizontal line), interquartile range (boxes), min/max values (whiskers), and outliers (circles). Statistically significant pairs are marked with horizontal bars (*p < 0.05). “High Gain” and “Low Gain” denote the disturbance conditions.

The summary results for the SR in disturbed trials are displayed in Figure 6. The performance for EMG and force feedback were similarly high in both delicate and power grasping when a low-gain disturbance was introduced in the control signal. In the case of high-gain disturbance, however, the subjects performed significantly better (*p* = 0.044) in power grasping with EMG feedback (73.3% {26.7}) than with force feedback (60% {20}). In delicate grasping, the median percentage for force feedback (33.3%) was also lower compared to EMG feedback (46.6%) but the difference was not significant.

The median number of repeated attempts ranged between 2 and 3 in all disturbed conditions, with no significant difference between the feedback types.

**FIGURE 7:**
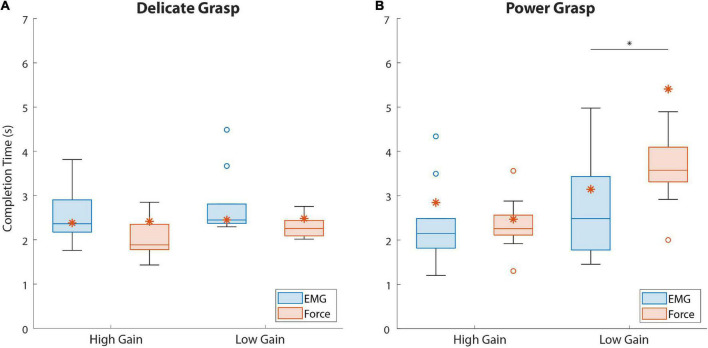
The last attempt completion time for both force targets. Boxplots **(A,B)** correspond to delicate and power grasps, respectively. The blue and red boxplots represent the summary performance with EMG and force feedback, respectively. The orange asterisks denote the performance of the amputee subject. The boxplots indicate the median (horizontal line), interquartile range (boxes), min/max values (whiskers), and outliers (circles). Statistically significant pairs are marked with horizontal bars (*p < 0.05). “High Gain” and “Low Gain” denote the disturbance conditions.

The results regarding the completion time of the last attempt in disturbed trials are shown in Figure 7. The completion time in delicate grasping was similar across all conditions (Figure 7A), with a median ranging between 1.9 and 2.5 s. In power grasping (Figure 7B), the results were similar for the two feedback types in the case of high-gain disturbance. However, in the low-gain disturbed trials the subjects using force feedback (3.64 s {0.73}) needed a significantly (*p* = 0.046) longer time to reach the target compared to those who used EMG feedback (2.48 s {1.66}).

The results regarding the amputee subject are denoted with asterisks in Figures 6, 7. Regarding the SR (Figure 6), the subject performed better with EMG feedback in three out of four conditions, with the largest difference in performance for delicate grasping and low-gain disturbance (93.3 against 73.3%). The SR for power grasping and high-gain disturbance was higher when EMG feedback was used (66.6 against 60%, Figure 6B) and the subject performed markedly faster in low-gain disturbed power grasp trials with EMG feedback (3.14 against 5.4 s, Figure 7B).

## Discussion

This study compared the performance of prosthesis grasping in a force-matching task with EMG and force feedback when the prosthesis control signal was disturbed. The disturbed trials, which appeared unbeknownst to the subjects and were interspersed with normal trials, were characterized by a gain that amplified or attenuated the control signal, thereby substantially altering the response of the prosthesis to the subjects’ muscle activity. Overall, the results imply the advantage of EMG over force feedback, albeit in fewer conditions than initially hypothesized.

We assumed that the prolonged training would allow the subjects to develop internal models for the control of the prosthesis and establish the mapping between the desired grasping force and the contraction required to achieve that force (Dosen et al., 2015c; Shehata et al., 2018). However, the internal models acquired during the training would not be valid for the disturbed trials. Since the force feedback only becomes available after the hand has grasped the object and produced force, the altered system dynamics would not be immediately obvious to the subjects and, consequently, the execution of the task would be compromised. Therefore, our assumption was that the subjects using force feedback would achieve lower performance in the disturbed trials, compared to those using EMG feedback. Specifically, the high gain disturbance was expected to lead to overshooting of the target force in the first trial and longer adaptation, while the low gain disturbance would prolong the completion time of the task in disturbed trials with force feedback.

Indeed, EMG feedback enabled the subjects to better react to the high-gain disturbance and achieve higher success in the first attempt. The ability to generate the correct force level despite the disturbance reduces the likelihood of accidents, such as slippage or breakage. However, the performance was significantly better only in the case of power grasping (Figure 6B). The slightly better baseline (non-disturbed) performance of EMG feedback in power grasping became, therefore, even more expressed in the presence of high-gain disturbances. Surprisingly, the subjects could not exploit the EMG feedback as effectively during delicate grasping, where we, in fact, expected the largest advantage. This is likely due to a combination of factors, including the narrower EMG range corresponding to level 2 (compared to level 4), high level of disturbance (doubled gain) and a limited amount of time available (until object contact) to correct the EMG based on the online feedback. And indeed, the subjects reported that they often became aware of the overshoot, but the hand had already closed around the block and applied a higher-than-desired force, at which point the subjects had to repeat the task.

In the case of low-gain disturbance, the two feedback types achieved similar SR for both grasp types. This can be attributed to the smoother myoelectric signal due to lower sensitivity and higher stability of the system resulting from the reduced gain. However, and following our initial assumption, the subjects indeed required a significantly longer time to complete a power grasp with force feedback than with EMG feedback (Figure 7B). Presumably, the subjects initially attempted to generate a contraction that they believed will be close to the target, based on their training. EMG feedback then allowed the subjects to immediately react to a lower myoelectric signal level and modulate upwards, even before the prosthesis contacted the object, thus increasing the prosthesis closing speed. On the other hand, when using force feedback, the subjects had to wait until the force was applied on the object to realize that their contraction was too low, at which point they used the feedback to gradually increase the force step by step, through the levels. Although slow, this strategy still allowed for good force control and disturbance compensation already in the first attempt (hence similar success rates as EMG feedback).

The similarity in the numbers of repeated attempts when using either feedback type in both grasp types was another surprising result. As mentioned before, force feedback was expected to underperform compared to EMG feedback, with the subjects adapting to the disturbances over a larger number of attempts per trial. There was, however, no significant difference in the performance of the two feedback types in disturbed trials. Given that the subjects needed only 1-2 additional attempts to adapt to the disturbance, the adaptation appeared not to have been difficult overall. This point, in combination with intrinsic feedback from the prosthesis, might be the reason for the lack of difference between the feedback types. Namely, even though the subjects did not have explicit information about the change in the control signal gain with force feedback (unlike with EMG feedback), they could estimate this indirectly, for instance, by observing the prosthesis motion (e.g., the prosthesis responding slower/faster than normal). Such incidental information might have been enough to drive the adaptation across repeated attempts. The use of incidental cues in prosthesis control has been reported in the past (Mann and Reimers, 1970; Prior et al., 1976) and investigated in more recent studies (Markovic et al., 2018b; Wilke et al., 2019; Gonzalez et al., 2021). These studies indeed showed that the subjects could accurately estimate the prosthesis closing velocity from visual observation of prosthesis motion (Wilke et al., 2019) and use this information together with natural muscle proprioception to control the grasping force (Markovic et al., 2018b).

Importantly, not only did the performance of the amputee subject follow the trends defined by the able-bodied subjects, but the EMG feedback seems to have been particularly beneficial in the case of the amputee. This is a promising result for the prospective clinical application of this approach. Moreover, the placement of the tactors on the upper arm did not have an impact on the quality of the feedback, since it remained intuitive and easily interpretable.

The gain disturbances used in the present study intend to simulate the changes in the amplitude of EMG signal that can arise during clinical applications (e.g., electrode shift or sweating) (Hahne et al., 2017; Yang et al., 2019). These factors can lead to either decreases or increases in the myoelectric signal amplitude (e.g., an electrode moving away from or closer to the signal source), and this corresponds to low and high gain disturbances of the present experiment, respectively. Nevertheless, the aim of this study was not to exhaustively investigate different types of signal changes but to demonstrate a fundamental insight, i.e., that different feedback types enable different disturbance compensation strategies. Other types of signal artifacts (e.g., transient changes), as well as disturbances during real-life uses, remain to be tested.

With EMG feedback, the subjects are likely to receive stimulation for a longer time (before and after contact) compared to force feedback (after contact only). However, the potential desensitization of the subjects due to prolonged constant stimulation was not assessed in the present study. Nevertheless, habituation is most pronounced when a prolonged stimulation is delivered to the same area on the skin (Buma et al., 2007). Considering that the EMG feedback is characterized by dynamic stimulation profiles (shift of active tactors) as well as the fact that the subjects can relax their muscles (no stimulation) after an object has been grasped (non-backdrivable prosthesis), we assume that desensitization will not be a serious limitation, even after prolonged use of a prosthesis equipped with EMG feedback. Indeed, none of the subjects reported difficulty in perceiving the feedback in the present experiment, despite the completion of many trials. Future studies could also be devoted to the systematic assessment of the subjective experience when using different feedback types.

Although the present study assessed the short-term adaptation to disturbance across successive trials, the potential learning over a longer time scale was not investigated. Nevertheless, it would be interesting to assess the evolution of disturbance compensation strategies as the user becomes increasingly familiar with closed-loop myoelectric control. Such assessment could elucidate if and how the type of feedback affects the learning over extended use.

The present study explored how the performance in a functional task with a myoelectric hand prosthesis integrated with EMG and force feedback differed in terms of response to disturbances in the prosthesis control signal. Overall, the results of this study indicate that, when a disturbance gain is introduced, the two feedback types perform similarly during the execution of delicate grasping, but EMG feedback provides a significant advantage in power grasps. A control scheme that displays better performance in the presence of a disturbed control signal is an important step toward a more reliable prosthesis-user interface.

## Data availability statement

The raw data supporting the conclusions of this article will be made available by the authors, without undue reservation.

## Ethics statement

The studies involving human participants were reviewed and approved by the Research Ethics Committee of the Nordjylland Region (approval number: N-20190036). The patients/participants provided their written informed consent to participate in this study.

## Author contributions

JT executed the data collection and analysis. All authors conceptualized the study, wrote the manuscript, and approved it for publication.
